# Malaria—Its Treatment

**Published:** 1906-07

**Authors:** Charles Hayes

**Affiliations:** Vinemont, Ala.


### MALARIA—ITS TREATMENT.

By CHARLES HAYES, M.D.,
VINEMONT, ALA.

The word malaria at one time meant bad air, but now, since the extensive works and investigations by Grassi, Manson, Koch, and others, the mosquito is the most common, if not the only, means of conveying the plasmodium to man; and only where they can grow and multiply can they cause symptoms of malaria in man.

If malaria ever spreads or infests those regions of the earth where snow and ice are always found, I suppose it would be proper for it to retain its original definition. The malarial plasmodium is found in some of the richest countries of the world, in fact, it seems that the malarial plasmodium is fond of rich soil. Whether this is of its own free will or to obtain the necessaries for the sustenance of its life, is not thoroughly known, but it is believed the latter is true.

It is generally conceded now that the mosquito is the common carrier of malaria and that the anopheles are the genus that the poison exists in, and of these there are more than four species. But I am quite sure we can have the anopheles without having malaria, for if the anopheles are healthy their bite will do no more harm than the culex.

But he must first obtain blood food from a human whose blood is impregnated with the malarial poison. The evolution of the plasmodium in the body of the mosquito is said to be about seven days (Osler), and throughout this time he is a sick mosquito; little does he believe as he draws the vital fluid from his victim that, at the same time, he is drawing into his stomach gametocytes which will soon give in return a great number of motile flagella. Here they grow and multiply until a sufficient number are matured, then they pass into the walls of his stomach, where they undergo changes similar to that in man.

So we see that the mosquito, according to experiments, suffers with gastritis, perhaps gastric ulcers, and may be many other things not yet worked out. Some say it is a true parasite of the red-blood corpuscles. It looks like, if that was true and it affects man and mosquito, it would get some of the other animals and insects. Some writers say that the horse, hog, cattle and insects are infected. But I believe most all these are immune except man and mosquitoes. At any rate, they are the two that give trouble. However the mode of infection and propagation may be, I believe man is the most common, and a far greater cause of the spread of malarial fever than the mosquito. For man can, under favorable circumstances, carry the poison a number of miles, while the mosquito carries it only a short distance, and as to elevation only a few yards. And mosquitoes work only at night.

Then, again, you sometimes see a malarial section free of malaria, but let some one come in this district whose blood contains the malarial plasmodium, and an epidemic of malarial fever is most sure to follow. The mosquito bites the infected man, hence they get the malarial plasmodium, and after it goes through a sufficient number of cycles to thoroughly inoculate him, his bite will have the same effect on man that he received when he bit the infected man.

In a case like this, I do not believe it is necessary to have the cesspools, swamps and decaying vegetation; all that is needed in a case like this is the mosquito and a temperature at or above 60° F, and the infected man. It has been strongly believed that man can be inoculated from drinking water impregnated with the malarial plasmodium. One case I recall of the three vessels of soldiers that crossed from Bona to Marselles. One of the vessel's water-supply was from a swamp, and on this vessel there was a severe outbreak of malarial fever, while the other two vessels, which had a pure water-supply, escaped without a single case. But very likely there were mosquitoes on these vessels, and possibly the impure water, if any amount of it was exposed to the mosquitoes, furnished a splendid breeding ground. But I believe it is just as important to protect the mosquitoes from man as man from the mosquito. I say this to emphasize the fact that man is sometimes the carrier of malarial plasmodium, and is often the cause of an epidemic.

But the most important part for us practitioners is to know how to diagnose and treat the various types of malaria. The most common type in the South is the tertian, though you frequently see the double tertian or quotitian.

There is no age that is exempt from this malady. It affects both young and old, rich and poor.

The diagnosis is very easy. You can usually make a diagnosis from the symptoms and locality of the patient.

Text-books say you can make a positive diagnosis with the microscope, but not all country doctors have laboratories equipped with microscopes.

When I am in doubt as to my diagnosis I put them on anti-malarial treatment, which will soon tell. The only disease it resembles here in North Alabama is typhoid fever, and the anti-malarial treatment does no harm during the first few days of typhoid fever.

And sometimes we have an attack of malarial and typhoid both.

In an attack of acute malarial you are not apt to see your patient until the time of the second or third stage. If during the second stage, he is very stupid and restless. He will tell you he feels bad, has dry skin, severe headache, may be sick stomach. His temperature will be anywhere from 104° F. to 106°. They will tell you they have been feeling bad for four or five days, and believe they had a light chill two days before that. And most all of them will tell you that they need liver medicine, their liver is not acting. If they are extremely restless I give a hypodermic of morphine and atropine. Then I give this:

℞ Dovers Powder.......... .......................... grs. x.
Sodium Salicylate....... .. ............... . ....... grs. x.
M. S. One every two or three hours till better.

I usually have this with me, and give them one myself. I then order:

℞ Quinine sulph........................................ ʒi.

Sodium Salicylate.................................... ʒiss.

Pul. Capsici.......................................... ʒss.

M. Ft. Cap. No. xii.

One every three hours.

I have many times warded off the second paroxysms with the above treatment.

As to diet, I usually let them have most anything they want, for they are not apt to want anything. I have them drink lemonade ad libitum. Also have them drink fruit juice, or most anything that contains acids. If they are anemic and need a tonic, after they have missed two or three chill days I give them:

℞ Fowlers Sol.......................................... ʒii.
Tr. Ferri Mur.......................................... ℥ss.
Syrup Quinine..........................(2 grs.) 2. s. ℥iv.
M. S. Teaspoonful after meals.

But it is not every time you have a typical case, and not every time they are free from complications.

The most common complications that I have had to contend with in my practice are:

Vomiting or sick stomach.

Hyperpyrexia.

Convulsions.

Pregnancy.

The rebellious stomach is usually due to a bilious condition of the system, which is very common in malarial subjects. For this I give one to two pints of warm water. This is usually vomited. Then I give 10 grs. calomel, with a little bismuth sub. nit. I divide this into four doses and give every hour, and follow this with castor oil. If they vomit the oil, I give them magnesium sulphate in hot lemonade, and by the time this acts the stomach will retain most anything I want them to have. If the stomach will not yet retain medicine, and if it is a severe case, I give quinine by inunction to defeat the next paroxysms. And for those patients with weak stomach I always use syr. quinine. This, in my practice, is tolerated better by those patients who become sick and vomit as the fever rises.

You would hardly think that hyperpyrexia would be a complication, but I had rather treat many of the complications than this one. In this class of patients with a very light chill you have following a very high fever. If they become excited their fever will run up. If they eat a little too much it will cause a marked rise of temperature. For the high fever I use cold water, usually wrap them in blankets wrung out of cold water. Then place them in bed and fold cover around them until they sweat freely. Convulsions are seen only in children. I believe it is caused from high fever or loaded bowel, or both.

For this I most invariably use gelsemium with a little aconite. You can give it in surprisingly large doses. I give them this until they are almost limber. In this way you can stop the convulsions, produce sweating, lower the temperature, and thus lessen the danger of cerebral congestion.

For children I use the tasteless syr. quinine. It has just as good effect and, I believe, is better tolerated by a weak stomach, either in children or adults. Of this syr. quinine I think Sharp and Dohme puts up the most palatable preparations of them all. Children don't mind taking it, which is another advantage.

Some physicians think pregnancy is a very serious complication in malarial fever. I do not consider it any complication at all, and only mention it because so many consider it a grave complication. Some physicians are afraid to give quinine to pregnant women, because of the believed oxytocic effect. This is a mistake, for you can give it with impunity to pregnant women. I have given it to them at most all stages of pregnancy, and have never had an abortion or miscarriage from its effects in my practice. I have given them five grain doses every three or four hours, when they had been pregnant six months, and many times under six months. I had one case last summer of five months' pregnancy. She had an attack of malarial fever the first time I saw her, her temperature was 102° F. I put her on three grain doses quinine every three hours. I saw her twenty-four hours later and her temperature was 105° F. I then ordered six grain quinine every three hours till fever went down. I saw her twenty-four hours later, and her temperature was 99° F. She recovered and went to term. Remember that continued high fever is dangerous in pregnancy. This treatment outlined is all I ever used in malarial fever. It is all I ever need. It is easy, simple and cheap, and I will say the last of these is important with those that dispense their own drugs, and do charity work. I will give the three drugs that you can cure malarial fever with: Quinine, Fowl. Sol. and Iron, named in the order of their importance. This treatment I have learned from four years experience. I treated forty-eight cases last autumn and never lost a case.

I have not mentioned pernicious malarial, because I know nothing about it. I never saw but one case, and it did not live long enough for me to learn much about it.

